# In-Situ Direct Synthesis of HKUST-1 in Wool Fabric for the Improvement of Antibacterial Properties

**DOI:** 10.3390/polym11040713

**Published:** 2019-04-19

**Authors:** Manuel J. Lis, Bianca Bastos Caruzi, Guilherme Andreoli Gil, Rafael Block Samulewski, Alesandro Bail, Fabio Alexandre Pereira Scacchetti, Murilo Pereira Moisés, Fabricio Maestá Bezerra

**Affiliations:** 1 Institute of Textile Research and Cooperation of Terrassa, Polytechnic University of Catalonia, C/Colom 15, Terrassa, 08222 Barcelona, Spain; manuel-jose.lis@upc.edu; 2 Textile Engineering, Federal University of Technology—Paraná, 635 Marcilio Dias St., Apucarana, 86812-60 Parana, Brazil; bi_caruzi@hotmail.com (B.B.C.); fabioscacchetti@utfpr.edu.br (F.A.P.S.); 3 Chemistry Engineering, Federal University of Technology—Paraná, 635 Marcilio Dias St., Apucarana, 86812-60 Parana, Brazil; guilherme_gil_@hotmail.com; 4 Núcleo de Inovação Industria (NI2), Federal University of Technology—Paraná, 635 Marcilio Dias St., Apucarana, 86812-60 Parana, Brazil; samulewski@utfpr.edu.br (R.B.S.); alebail@utfpr.edu.br (A.B.); murilomoises@utfpr.edu.br (M.P.M.)

**Keywords:** HKUST-1, wool, antibacterial

## Abstract

The use of Metal-Organic Frameworks (MOF) such as HKUST-1 in textiles is an alternative with regard to the development of technologies that are increasingly seeking for functionalities, mainly in the fields of health and hygiene, named biofunctional fabrics. However, the application of the MOF under the surface of the wool fiber can lead to a low durability finish due to its low fixation. Thus, this project aims to perform the direct synthesis of HKUST in the wool fiber, so that a product with good washing durability can be obtained. The purpose of this study was to incorporate metal-organic frameworks, composed of copper and trimesic acid, into woolen fabrics, to improve the antibacterial properties. The synthesis was performed directly in the wool fabric, at time intervals of 24 and 48 h. The resulting fabrics were characterized by Scanning Electron Microscopy (SEM), Energy Dispersive Spectroscopy (EDS), X-Ray Diffractometry (XRD), Fourier Transform Spectroscopy Infrared-Attenuated Total Reflectance (FTIR-ATR), and colorimetric analysis (CIElab), and the Antimicrobial Activity Test (American Association of Textile Chemist and Colourists - AATCC Test Method 61-2007-2A) was performed. The results suggested that the application produced textiles with antibacterial properties, showing activity against *Escherichia coli*.

## 1. Introduction

Textiles with improved functionalities can find a variety of applications, such as controlled release of drugs [1,2,3], cosmeotextiles [1,4,5], temperature control [6,7], and anti-microorganism action [8,9,10]. The use of antimicrobial agents in textile items is focused on the control of infections coming from the contact between the textile and human skin [11,12]. Currently, the treatments to setup the antibacterial fibers using metallic salts aim at the incorporation of silver [13], copper [6,14], and zinc [15,16] nanoparticles.

Among those, the use of copper as an antimicrobial agent stands out because of its low cost [8]. However, the durability effect is restricted because of the low interaction between the nanoparticles and the textile. In this sense, an alternative for a better finishing is to synthesize the metal into a structure that can interact with the textile. One of the possibilities of this alternative is the synthesis of Metal-Organic Frameworks (MOFs), which are crystalline solids formed by organic ligands and inorganic agglomerates, as metallic salts, that create a highly-porous three-dimensional structure [17,18,19,20].

Since the MOFs are formed by organic and inorganic parts, they present high adsorption capacity, which is provided by the organic precursors and a highly-ordered structure coming from the inorganic ones [20]. Yet, the metal present in the structure may contribute with new desired effects [12]. Thus, the immobilization of MOFs in a textile substrate becomes an alternative for the combination of the porosity present in the MOF and the properties of the metals, resulting in a synergistic effect that enhances their durability [14,21]. 

The use of MOFs on the textile surface can be reached either by modification or direct application, having direct dependence on the kind of fiber and MOF, due to the chemical groups present on the surface of the fiber [22,23,24]. 

Some experiments carried out in textile substrates were MIL-101 (Matériauxs de L’Institut Lavoisier-101) in polyamide [25], which aimed to dye the textile, MOF-100 in cotton [26], which enhanced antimicrobial properties, MOF-5 in silk [27], which removed dyes, and MOF-199 in wool [22] and polyacrylonitrile fibers [24] that aimed at the removal of fuels and methyl parathion. 

Since it was possible to synthesize those structures in textiles, the present paper aims to incorporate an MOF, HKUST-1 (MOF-199), in woolen articles in a direct way for antibacterial functionalization, since the wool fiber acts as an agent of the propagation and hosting of bacteria [28]. Thus, it is intended to develop a new antimicrobial finish for woolen fabric from the direct incorporation of MOF.

## 2. Materials and Methods

All reagents were of analytical-grade purity: trimesic acid (TMA) (Sigma-Aldrich, 98%, São Paulo-SP, Brazil), copper (II) nitrate (Vetec, 98%, Duque de Caxias, RJ, Brazil), and N,N-dimethylformamide (DMF) (Vetec, 98%). The textile substrate consisted of standard wool fabric (ISO 105-F06), approximate weight: 125 gm^−2^, Test Fabrics Inc. (Swedesboro, NJ, USA).

### 2.1. MOF Composite Synthesis 

Direct incorporation of HKUST-1 into fabrics was carried out according to a modified method [29]. In the first step, two solutions of 250 mL of DMF (73.09 g/mol) were prepared. For the first solution, 18 g of copper nitrate were added, and in the second solution, 9 g of TMA were added, both under stirring. As soon as both reagents were dissolved, they were mixed together, resulting in a single solution. 

Each wool sample of 1 g was inserted into 50 mL of solution inside an oven (85 ± 2 °C). At the end of each interval (24 and 48 h), the samples were removed from the heater, washed with DMF, and dried at room temperature. Afterward, the pure HKUST-1 was prepared by adding 4 g of copper nitrate and 2 g of TMA into 50 mL of DMF solution *(*(85 ± 2 °C) during 20 h, after which they were washed with DMF and dried at room temperature.

### 2.2. Characterization of HKUST-1

The analysis of the thermal stability of the MOF was performed using the thermogravimetric equipment TGA.SDTA851-Mettler Toledo (Barueri, SP, Brazil) and the Software *S*TARe (Version SW 9.01). The method employed used a heating rate of 10 °C·min^−1^ and a temperature range from 30–800 °C in an atmosphere of nitrogen.

### 2.3. Characterization of Textile Finishing (Wool@MOF)

For the morphological, structural and molecular analysis of the materials (wool@MOF), a Scanning Electron Microscope (SEM) (Model Quanta 250, Waltham, MA, USA), an Energy Dispersive Spectroscopy (EDS) ( x-act model, Waltham, MA, USA) and the AZtec 3.0 SP2 software provided by Oxford Instruments, an X-Ray Diffractometer (PXRD) (Shimadzu Model XRD-6000, Tokyo, Japan), and a Fourier Transform Spectroscopy Infrared-Attenuated Total Reflectance (FTIR-ATR) (Frontier-Perkin Elmer, São Paulo, SP, Brazil) with a diamond ATR attachmen, were used. Fabric thickness measurements, in triplicate, were taken for untreated, treated (24 and 48 h), and after washing, using the micrometer. The PXRD was carried out using a copper irradiation tube operating at 40 KV and 30 mA in the region from 5 (2θ°) to 40 (2θ°) with a dwell time of 2θ°/min. The FTIR spectrum was evaluated with 1 cm^−1^ and 64 scan accumulations, with the range in the infrared spectrum between 650 and 4000 cm^−1^. The techniques (SEM, PXRD, and FTIR-ATR) were carried out on the wool fabrics with and without the finishing.

The durability with respect to washing of the functionalized wool was verified by SEM after subsequent wash cycles (5 washes). Washing was carried out in accordance with the AATCC Test Method 61-2007-2A.

The antibacterial properties were tested in both untreated and treated fabrics. The test was performed against *E. coli* bacteria ASTM E2149-13a: Standard Test Method for Determining the Antimicrobial Activity of Immobilized Antimicrobial Agents under Dynamic Contact Conditions. 

The CIE color coordinates (L*, a*, b*) for the treated and washed fabrics were obtained, all samples were measured in three independent areas, using a spectrophotometer Delta Vista 450G and the i7 Delta Color software, adjusting to 10° for the observer with D65 illuminant, visual geometry of d/2, and 2 mm of measurement area. The color coordinate parameters were lightness (L*) from black to white (0–100), a* the red/green ratio (+/−), and b* the yellow/blue ratio (+/−) [30].

## 3. Results and Discussion

### 3.1. Study of the HKUST-1 Synthesis

**Figure 1:**
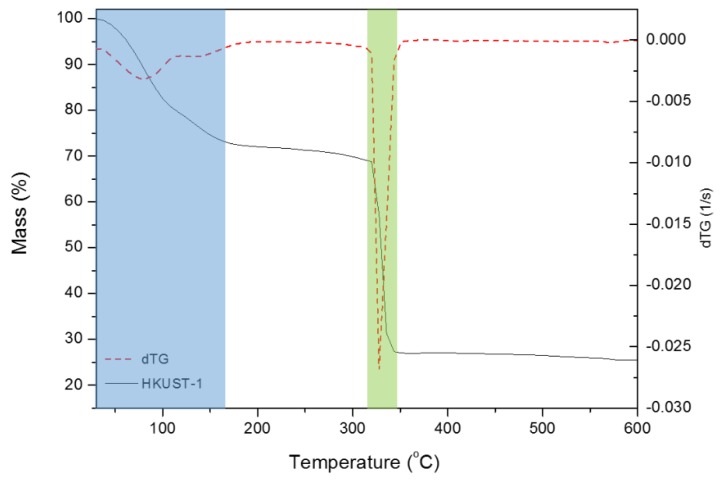
Thermogravimetric curves of HKUST-1.

Figure 1 presents the mass loss of pure compounds HKUST-1, heated from 30–800 °C in an atmosphere of nitrogen at the rate of 10 °C per minute. The TG and dTG curves were similar to the HKUST-1 [31]. The first stage of mass loss occurred between 30 and 164.4 °C (dehydration of the HKUST-1), indicating moisture of 26% (mass per mass ratio, m:m). Stage 2 of mass loss occurred from 322.2 and 345.9 °C (direct degradation of the HKUST-1 material) with high thermal stability due to large crystalline molecular order, high interaction metal clusters/linkers, and high structural purity [32]. The data obtained in this analysis resembled the results of Lin and Hsieh [33] and Rizwan et al. [34]; HKUST-1 decomposed at approximately 300 °C, with a 50% mass loss. In this work, the mass loss was 43%, showing that the compound analyzed was HKUST-1 and confirmed by PXDR analysis.

### 3.2. Study of the Synthesis on the Surface of the Wool

**Figure 2:**
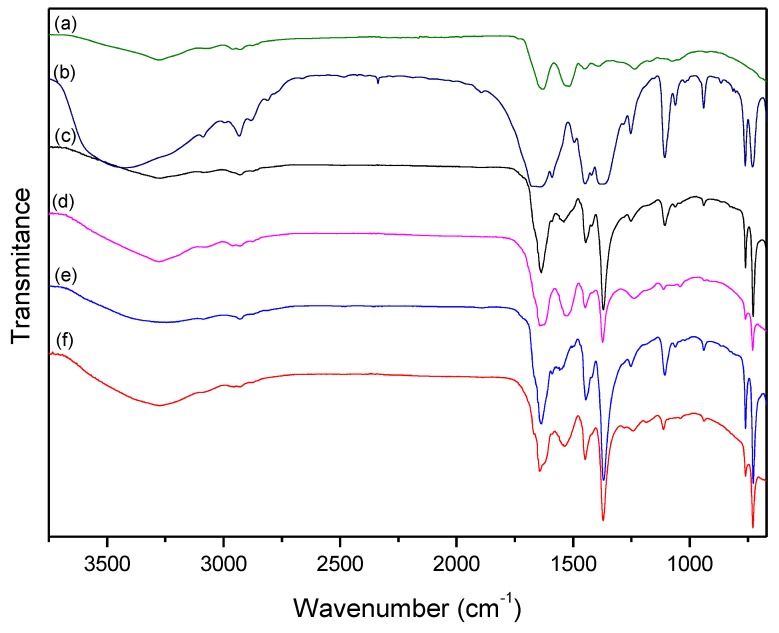
FTIR-ATR of: (**a**) woolen fabric (untreated); (**b**) HKUST-1; wool@HKUST-1 synthesis of: (**c**) 24 h; (**d**) 24 h washed; (**e**) 48 h; and (**f**) 48 h washed.

Figure 2 illustrates the FTIR spectra of treated and untreated samples. These IR spectra show signals of wool and HKUST-1. 

HKUST-1 FTIR, Figure 2b, spectra exhibited MOF formation bands [35,36]; see Table 1. Pure keratin comprised 90% of the wool, yielding the behavior of the FTIR for a protein. According to Figure 2a, wool FTIR spectra coincided with keratin polymer bands [37]; however, the spectra also showed bands at 1735, 1453, and 1393 cm^−1^ consistent with terminal COOH and NH_2_ functionalities. These bands allow the wool to serve as a prospective anchoring fiber for the growth of HKUST-1 [38,39].

Due to the low pH used in the synthesis, the protonated groups will not coordinate directly with copper. As the MOF/fabric and samples after treatment spectra nearly corresponded, identifying the fabric group responsible for MOF anchoring is unlikely. Studies have shown that acid and oxidant treatments (such as the presence of nitrate and pH 3 used in this work) break S–S bonds and form new terminal functional groups such as –SH, –SO–S–, and SO_3_– [40,41,42,43]; consequently, new bands, absent in untreated wool, appear in the region between 1000 and 1120 cm^−1^. When observing the spectra of the treated and washed fabric, with low superficial MOF loss, small bands appear in the region between 1000 and 1120 cm^−1^, and the intensity at 3400 cm^−1^ increases, indicating S–S bond cleavage [44].

According to Zhang et al. [45], S–H functional groups and increased fabric hydrogen bonds affect these regions of the FTIR spectra. Therefore, the observation of the FTIR disorients the identification of the functional group related to MOF anchoring. In addition, the literature shows that the acid treatment increases the number of coordination sites proportionally to the hydrogen interactions; and the absence of shifts for C=C aromatic vibration frequencies, pointing to the lack of the influence of the aromatic ring in the MOF–wool interaction [21].

**Figure 3:**
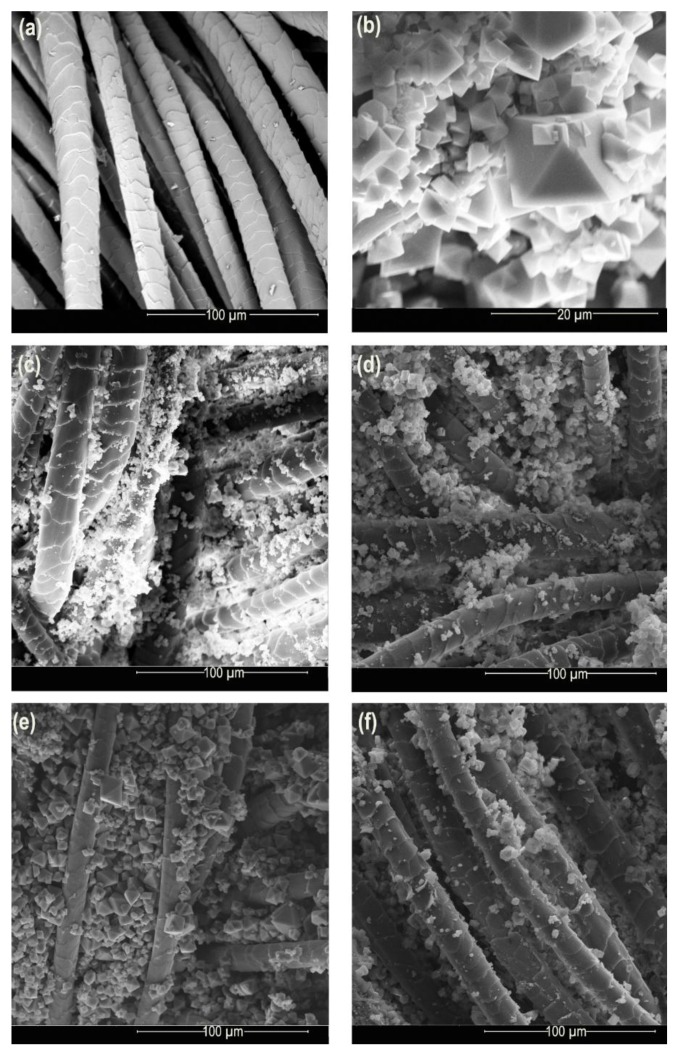
SEM of: (**a**) woolen fabric (untreated); (**b**) HKUST-1; wool@HKUST-1 synthesis of: (**c**) 24 h; (**d**) 24 h washed; (**e**) 48 h; and (**f**) 48 h washed.

The observation of FTIR spectra for all samples, in Figure 2d,f, allows concluding that washing samples reduced the intensity of HKUST-1 bands and removed MOF from the wool. SEM images of all samples, Figure 3, show the same amount of MOF particles, demonstrating that copper and terminal function linking are non-exclusive interactions between MOF; non-covalent weak interactions like hydrogen binding occur. 

Figure 3 displays the surface morphology of wool with the incorporation of HKUST-1 with visible nanoparticles on the fiber before and after washing. 

**Figure 4:**
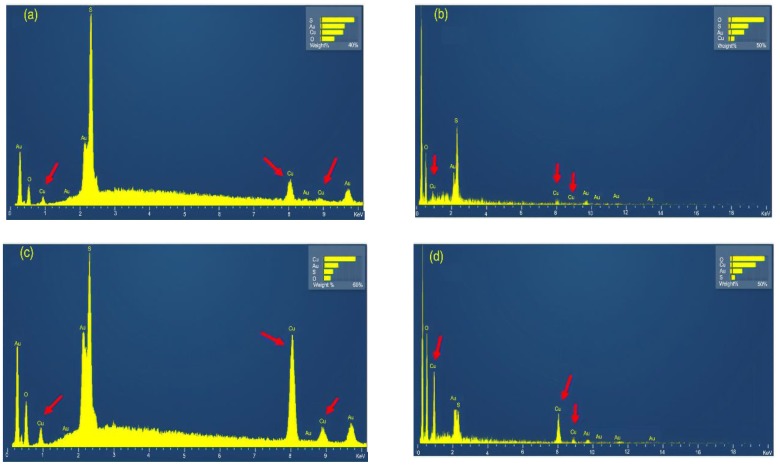
EDS of wool@HKUST-1 synthesis of: (**a**) 24 h; (**b**) 24 h washed; (**c**) 48 h; and (**d**) 48 h washed.

The morphology of the MOFs, Figure 3b, resembles an octahedral structure, as presented by Lin and Hsieh [33], Hosseini, Zeinali, and Sheikhi [46], and Toyao et al. [47]. Figure 4 compares the EDS spectra of wool@HKUST-1 prepared for 24 h (a), 24 h after washing (b), 48 h (c), and 48 h after washing (d). These spectra reveal that the metal organic framework HKUST-1 was added on the wool fibers’ surface, and its presence after washing was proven by cooper signals (near 1.0, 8.0, and 9.0 KeV). These results indicate the performance of the synthesis on the surface of the fabric, as indicated by the thermal analysis and PXRD.

**Figure 5:**
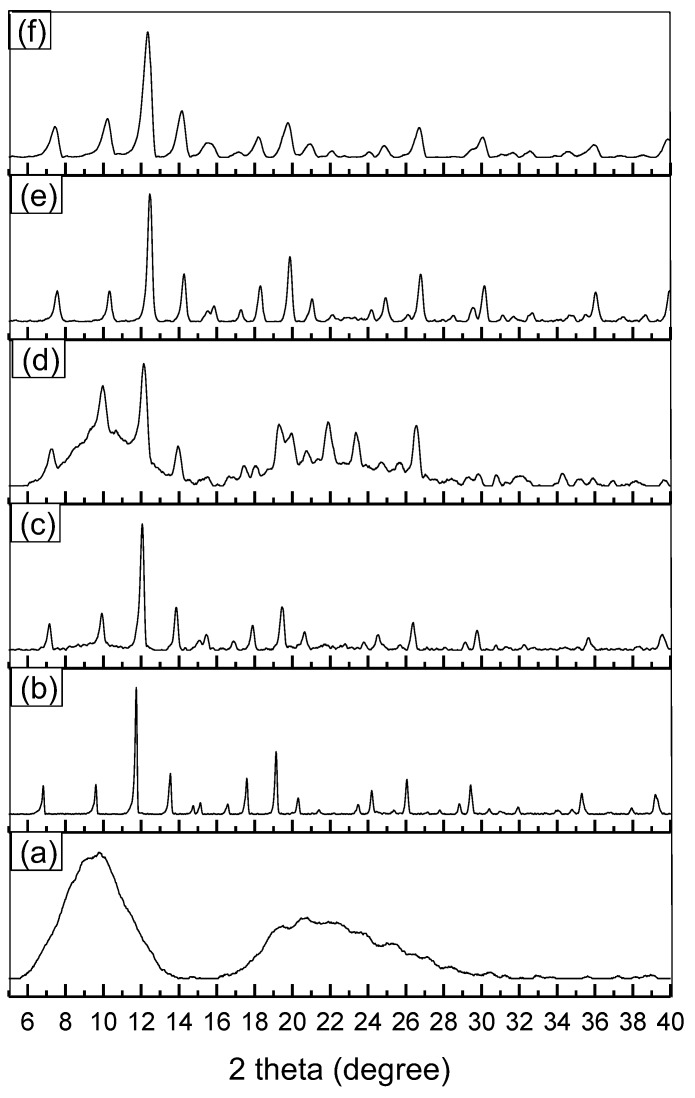
PXRD of (**a**) woolen fabric (untreated); (**b**) HKUST-1; wool@HKUST-1 synthesis of: (**c**) 24 h; (**d**) 24 h washed; (**e**) 48 h; and (**f**) 48 h washed.

PXRD analysis determined the crystallinity of the structures and confirmed the presence of HKUST-1 on the surface of the wool. Figure 5 shows the diffractograms of non-treated wool fabric, MOF HKUST-1 (conventional method), and treated wool before and after washing. A direct relation is observed concerning time and the presence of HKUST-1 on the surface. The charts for wool and MOF, shown in Figure 5, exhibit two characteristic peaks, 2θ ≈ 10° for the wool and 2θ ≈ 13° for the HKUST-1. 

The diffractogram of synthesized MOF in [28] and [41] shows the same peaks, especially the one at 2θ ≈ 13°. In general, before the washing, the behavior was similar to the MOF diffractogram (a), with sharp peaks close to 2θ ≈ 13° and 2θ ≈ 20°’; however, once samples were washed, as in the diffractogram in (d), they showed similar wool peaks in the 2θ ≈ 10° region and from the 20 to 2θ ≈ 25° range, resulting from the fabric peaks overlap of HKUST-1, Figure 5c,d. This difference shows that washing causes a slight decrease in the number of crystalline structures over the fabric.

Nevertheless, Figure 5e,f shows the contrary. After 48 h of synthesis, the washing effects decreased, with more MOF observed on the surface of the fabric. In general, it is possible to see the peak at 2θ ≈ 13° indicating effective incorporation of MOF.

### 3.3. Evaluation of the Finish

The color analysis verified the presence of HKUST-1 on the wool surface; copper present in the fabric changed its color from a blue to a green hue. Table 2 shows the color data before and after samples were washed. The values of L*, a*, and b* refer to the luminosity, coordinates red/green, and coordinates yellow/blue, respectively.

The luminosity value of the untreated sample was 83.94 ± 0.12. Regarding the values of the treated samples before and after washing (in this order), the 24 h synthesis presented the values: 56.35 ± 0.67 and 54.25 ± 0.32; the 48 h synthesis presented the values: 55.81 ± 0.41 and 48.55 ± 0.29. This reveals that the samples lost luminosity, getting darker because of the MOF presence [6]. MOF and fiber macromolecules interacted and changed the color of the fiber from blue (Cu^2+^) to green: coordination of copper (II) with the functional groups of wool. The absorption band shifted from the visible to near-infrared region explained by (1) HKUST-1 water ligand substitution from wool functional groups, such as carboxyl and thiol, and MOF–wool interaction removing electronic density from copper (II), increasing the ionic character. Thus, Ligand-Metal Charge Transition (LMCT) shifted to lower energy regions. (2) There was a decrease in the Crystal Field Stabilization Energy (CFSE) due to the substitution of water ligand possibly by wool functional groups, once they displayed more intense-donors than the first [48,49]. 

**Figure 6:**
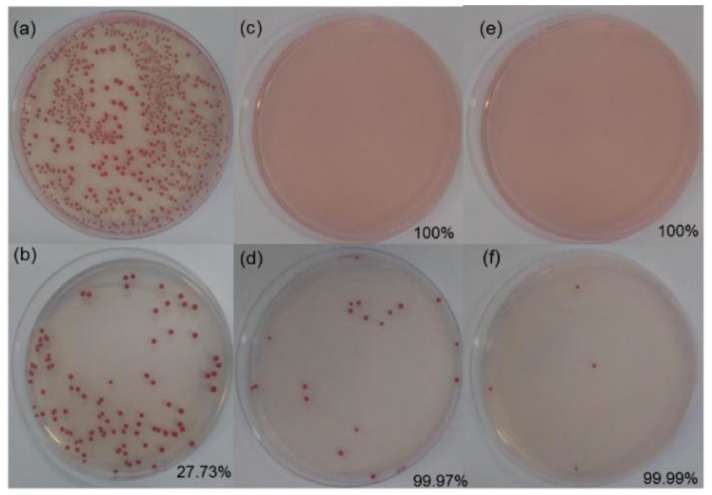
Antimicrobial activity (reduction of *E. coli* under dynamic contact conditions): (**a**) positive control; (**b**) woolen fabric; wool@HKUST-1 synthesis of: (**c**) 24 h; (**d**) 24 h washed; (**e**) 48 h; and (**f**) 48 h washed.

Figure 6 shows the antimicrobial activity for untreated and treated wool fabric before and after washing.

The untreated woolen fabric and the positive control showed an expected growth of microorganisms, and the untreated fabric had a reduction of 27.73%, as shown in Table 3.

The presence of MOF in the treated samples attributes them antimicrobial effects, as presented by Wyszogrodzka et al. [12]. The presence of copper in the MOF structure eliminates microorganisms due to the intrinsic property of this metal. The wool@HKUST-1 fabric completely inhibited the tested microorganism. The results after washing showed a slight decrease in the level of inhibition of growth of the microorganisms; nevertheless, the inhibition remained almost complete: 99.97% after 24 h and 99.99% after 48 h of treatment. The fabrics treated with HKUST-1 during the synthesis of 48 h are noteworthy, with better antimicrobial performance.

## 4. Conclusions

The present work verified the growth of metal-organic structures on woolen fabric. The SEM, PXRD, and color analyses revealed the increasing amount of those structures on the fabric with the influence of the time of synthesis. Furthermore, they proved that those crystalline solids were not just deposited, but linked to the wool, since the samples showed the presence of copper on the surface able to completely inhibit the microorganism *E. coli*, even after washing.

## Figures and Tables

**Table 1:** FTIR-ATR bands’ assignment for HKUST-1, wool, and treated wool samples.

HKUST-1		Wool		Treated		
Band cm^−1^	Assignment	Band cm^−1^	Assignment	Bandcm^−1^	Assignment	Corresp.
3430	υO–H (water)	3277	υN–H	3255	υN–H	HKUST-1
2930	υC–H	2995	υC–H	2930	υC–H	HKUST-1
1659	υ_s_COO^−^	2928	υC–H	1719	υCOOH(term.)	Wool
1590	υ_as_COO^−^	1735	υCOOH(term.)	1650	υ_s_COO^−^	HKUST-1
1453	δ_a_C–O	1634	υC=O (amide)	1634	υC=O (amide)	Wool
1375	υC=C (aromatic)	1524	δN-H + υC–N	1590	υ_as_COO^−^	HKUST-1
1252	υC=C (aromatic)	1453	υN-H (term.)	1550	δN–H + υC–N	Wool
1107	δC–H (ip)	1393	υC-O (term.)	1445	δ_a_C–O	HKUST-1
935	δC–H (oop)	1237	υN–H + δC–N	1368	υC=C (aromatic)	HKUST-1
494	υCu–O			1252	υC=C (aromatic)	HKUST-1
				1107	δC–H (ip)	HKUST-1
				938	δC–H (oop)	HKUST-1

υ = stretching vibration; υ_s_ = symmetric stretching vibration; υ_as_ = asymmetric stretching vibration; δ_a_ = axial deformation; δ = bending; ip = in plane; oop = out of plane; term. = terminal; Corresp. = correspondence of the band.

**Table 2:** Color data for the wool fabric modified with HKUST-1 after 24 and 48 h of direct synthesis.

Synthesis Time	L*		a*		b*	
	No Wash	Washed	No Wash	Washed	No Wash	Washed
Untreated	83.94 ± 0.12		−0.47 ± 0.08		11.68 ± 0.15	
24 h	56.35 ± 0.67	54.25 ± 0.32	−41.64 ± 0.53	−25.20 ± 0.55	4.60 ± 0.63	17.52 ± 0.66
48 h	55.81 ± 0.41	48.55 ± 0.29	−42.43 ± 0.47	−21.76 ± 0.31	−4.44 ± 0.49	15.56 ± 0.52

**Table 3:** Antimicrobial activity in treated and untreated wool fabrics.

Sample	No. of *E. coli* at Zero Time	No. of *E. coli* Final	Reduction (%)
Untreated	6.30.10^5^	1.75.10^5^	27.73
24 h	6.30.10^5^	0	100
24 h/washed	6.30.10^5^	1.70.10^2^	99.97
48 h	6.30.10^5^	0	100
48 h/washed	6.30.10^5^	4.00.10^1^	99.99
